# Outcomes from a cohort of patients with acute kidney injury subjected to continuous venovenous hemodiafiltration: The role of negative fluid balance

**DOI:** 10.1371/journal.pone.0175897

**Published:** 2017-04-20

**Authors:** Thais Oliveira Claizoni dos Santos, Marisa Aparecida de Souza Oliveira, Julio Cesar Martins Monte, Marcelo Costa Batista, Virgilio Gonçalves Pereira Junior, Bento Fortunato Cardoso dos Santos, Oscar Fernando Pavão Santos, Marcelino de Souza Durão Junior

**Affiliations:** 1 Nephrology Division, Universidade Federal de São Paulo, São Paulo, SP, Brazil; 2 Nephrology Division, Hospital Israelita Albert Einstein, São Paulo, SP, Brazil; University of Sao Paulo Medical School, BRAZIL

## Abstract

**Background:**

Several factors influence the outcomes in acute kidney injury (AKI), especially in intensive care unit (ICU) patients. In this scenario, continuous renal replacement therapies (CRRT) are used to control metabolic derangements and blood volume. Knowing this fact, it may be possible to change the course of the disease and decrease the high mortality rate observed. Thus, we aimed to evaluate the main risk factors for death in AKI patients needing CRRT.

**Results:**

This was a prospective, observational cohort study of ICU patients (N = 183) with AKI who underwent continuous venovenous hemodiafiltration (CVVHDF) as their initial dialysis modality choice. The patients were predominantly male (62.8%) and their median age was 65 (55–76) years. The most frequent comorbidities were cardiovascular disease (39.3%), hypertension (32.8%), diabetes (24%), and cirrhosis (20.7%). The main cause of AKI was sepsis (52.5%). At beginning of CVVHDF, 152 patients (83%) were using vasopressors. The median SAPS 3 and SOFA score at ICU admission was 61 (50–74) and 10 (7–12), respectively. The dialysis dose delivered was 33.2 (28.9–38.7) ml/kg/h. The median time between ICU admission and CVVHDF initiation was 2 (1–4) days. The median cumulative fluid balance during the CVVHDF period was -1838 (-5735 +2993) ml. The mortality rate up to90 days was 58%. The independent mortality risk factors in propensity score model were: chronic obstructive pulmonary disease (OR = 3.44[1.14–10.4; p = 0.028]), hematologic malignancy (OR = 5.14[1.66–15.95; p = 0.005]), oliguria (OR = 2.36[1.15–4.9; p = 0.02]), positive daily fluid balance during CVVHDF (OR = 4.55[2.75–13.1; p<0.001]), and total SOFA score on first dialysis day (OR = 1.27[1.12–1.45; p<0.001]).

**Conclusions:**

Dialysis-related factors may influence the outcomes. In our cohort, positive daily fluid balance during CRRT was associated with lower survival. Multicenter, randomized studies are needed to assess fluid balance as a primary outcome to define the best strategy in this patient population.

## Introduction

Acute kidney injury (AKI) is common in patients admitted to intensive care unit (ICU) and is associated with the dysfunction of multiple organs and systems (DMOS) [1]. These patients are generally older people [2, 3], presenting with multiple comorbidities and some degree of renal dysfunction prior to hospitalization, and have often undergone invasive procedures [4]. The main cause of AKI is sepsis, which accounts for approximately half of the cases [4–6].

A significant number of these patients require dialysis during disease development. Within this setting, in which patients present with hemodynamic instability, are on mechanical ventilation, and are hypercatabolic and oliguric, continuous renal replacement therapies (CRRT) are often used [4].

In such cases, several factors influence the outcome. Some of these variables are related to the intrinsic conditions of the patient, such as age, comorbidities, severity of the disease that caused the AKI, metabolic disorders [4, 7], organ dysfunctions, and weight gain during the resuscitation phase [8, 9]. Other factors that seem to influence the prognosis of these patients are those associated with the therapy itself, such as the type of dialysis modality [10], the time when the procedure was initiated [5], the delivered dose of dialysis, metabolic control and volume status obtained during the course of treatment [11].

Fluid resuscitation is considered a cornerstone therapy for critically ill patients at risk for or with AKI [12], especially with the purpose to maintain adequate renal perfusion, to treat hypovolemia, and to improve cardiac output [13,14]. Recently, the concept that liberal fluid administration brings positive consequences for the kidney has been challenged. Several observational studies have linked positive fluid balance with AKI occurrence and mortality, especially before RRT initiation [8, 14–16]. However, only a small number of studies have evaluated the association between fluid overload and mortality during renal replacement therapy [11,15,17, 18].

Our aim was to assess the factors related to death in a cohort of patients admitted to the ICU with AKI in need of dialysis, who underwent continuous venovenous hemodiafiltration (CVVHDF) in a private and a tertiary hospital.

## Materials and methods

### Type of study

A prospective, observational study.

### Study setting

The study was conducted in the adult ICU of the Hospital Israelita Albert Einstein (HIAE), in Sao Paulo (SP), Brazil. The recruitment period was from January 20, 2013 to April 30, 2014.

### Population

#### Inclusion criteria

The study included ICU patients with AKI, who had been initially referred for CVVHDF by the nephrology medical team.

#### Exclusion criteria

Patients who refused to participate in the study.

Patients diagnosed with end-stage renal disease (ESRD) and already undergoing regular dialysis program.

Patients who did not complete 24 hours of therapy.

### Parameters analyzed

The following baseline characteristics were assessed: age, gender, race, comorbidities, such as diabetes mellitus (DM), hypertension, cardiovascular disease (CVD), chronic obstructive pulmonary disease (COPD), cirrhosis, neurological diseases, rheumatic diseases, solid tumors, hematologic malignancies, bone marrow transplantation (BMT), acquired immunodeficiency syndrome (AIDS), and solid organ transplant.

The study also assessed the reasons for ICU admission, the type of patient (medical or surgical), and the main causes of AKI.

The SAPS 3 score (Simplified Acute Physiology Score 3) was used as an indicator of severity and prognosis while the patient was in the ICU. The SOFA score (Sequential Organ Failure Assessment) was used to determine organ dysfunctions and was assessed on admission to the ICU and on the first (D1), third (D3), and seventh (D7) days of CVVHDF.

On initiating CVVHDF, we assessed the use of vasopressors and/or inotropic agents, mechanical ventilation, sedation, and enteral and parenteral nutrition, as well as the presence of oliguria (diuresis <400 ml/24 hours in the first CVVHDF day).

Changes in body weight (%) were also analyzed during the period between hospital admission and CVVHDF initiation.

### Baseline renal function and AKI stages

Baseline renal function was assessed through the lowest levels of plasma creatinine within a 3-month period before admission (when available), or levels at hospital admission. The corresponding glomerular filtration rate (GFR) was estimated through the formula of the Modification of Diet in Renal Disease (MDRD) study.

On initiation of dialysis, the AKI was classified according to the KDIGO (Kidney Disease: Improving Global Outcomes) guidelines. Briefly, the AKI is classified as stage 1 when there is an absolute increase of 0.3 mg/dl in creatinine levels or an increase greater or equal to 150 to 200% of the creatinine level or a urinary flow lower than 0.5 ml/kg/h for more than 6 hours. Stage 2 is reached when creatinine levels are greater than 200 to 300% or the urinary flow is lower than 0.5 ml/kg/h for a period of more than 12 hours. Stage 3 is reached when creatinine levels increase to over 300% or higher than 4.0 mg/dl, with an increase of at least 0.5 mg/dl, a urinary flow lower than 0.3 ml/kg/h for 24 hours, or anuria for 12 hours.

### Dialysis data

The following CVVHDF data were investigated: the time lapse between ICU admission and initiating dialysis, the dialysis dose (in ml/kg/h of effluent), the duration (in days) of treatment, fluid balance (FB) (in L) during CVVHDF period, and daily fluid balance (L). The FB was calculated from the difference between losses (diuresis, drains, loss by dialysis) and gains (nutrition, fluids, vasopressors, and other medicines). The delivered dose of dialysis was calculated daily by multiplying the volume of effluent by the ratio of the urea effluent and serum urea.

### Outcomes

Mortality rates were assessed for up to 90 days after initiating CVVHDF. For surviving patients, dialysis dependence was assessed at the time of hospital discharge, and renal function (in those who had recovered it) was determined by measuring creatinine levels and the estimated glomerular filtration rate (eGFR).

### Treatment protocol

The protocol was based on the one performed in a previous study [19]. Briefly, CVVHDF was performed with a Prismaflex machine (Gambro Renal Products, France), with an M100 hemofilter and an AN69 membrane (Gambro Renal Products, France).

Vascular access was obtained by inserting an 11.5 F triple-lumen venous catheter (Arrow International, PA, USA) into the internal jugular vein (preferably on the right side), or into the femoral vein, guided by ultrasound. Blood flow was maintained constant at 100 ml/minute.

Regional citrate anticoagulation was applied with a 4% trisodium citrate solution and calcium replacement solution (0.75% CaCl2) were infused into the arterial line (through the three-way tap) and into the third line of the dialysis catheter, respectively. The infusion of citrate solution was adjusted by the nursing staff according to a spreadsheet to maintain the concentration of post-filter ionic calcium within a range of between 0.25 and 0.30 mmol/L. Similarly, the calcium solution infusion was adjusted to maintain the systemic concentration of ionic calcium within a range of between 1.12 and 1.20 mmol/L.

The prescribed dialysis dose was 35 ml/kg/h (total volume of effluent).

The hemofilter and the extracorporeal circuit were replaced every 72 hours.

### Ethics approval and consent to participate

Patients or the responsible caregiver received and signed the informed consent forms. The project was approved by the Local Ethics Committee (Hospital Israelita Albert Einstein), under number 1627–12.

### Statistical analysis

The qualitative variables were described in absolute frequencies and percentages, and the quantitative variables were described in means and standard deviations or medians and interquartile ranges (IQR), depending on the distribution of normality. The distribution of numerical variables was investigated through histograms and the Shapiro-Wilk test of normality. The survival function within 90 days was estimated by the Kaplan-Meier method.

The association between categorical variables was evaluated by Pearson’s chi-squared test or Fisher’s exact test. Comparisons between numerical variables were performed by t-test or Mann-Whitney’s test, depending on normality.

An analysis of factors associated with death was performed through simple and multiple forward logistic regression models. Variables with a p-value less than or equal to 0.15 in the simple approach were included in a multivariate model, in which the variables were selected step-by-step. After selecting the variables, only the final obtained multiple models was presented. The results of models were presented in estimated odds ratios and 95% confidence intervals as well as the p-values. An additional Kaplan Meier survival analysis was performed within 90 days of CVVHDF initiation with each variable related with mortality and comparisons were made using the Log Rank method.

We performed additional statistical analysis including Cox proportional hazard model and a propensity score analysis to adjust for confounds. The propensity score was estimated by using a multivariate logistic regression of patients receiving positive mean daily fluid balance or not. Thus, the propensity score analysis was maintained as final model. The Cox regression analysis was included as supplementary file.

Analyses were performed using the R package (*R Core Team*, *2013*. *R*: *A language and environment for statistical computing*. *R Foundation for Statistical Computing*, *Vienna*, *Austria*. *URL http://www.R-project.org/*). The significance level was 5%.

## Results

### Description of the demographic and clinical characteristics of patients

A total of 207 patients were assessed, of whom 7 were excluded because they did not complete twenty four hours of therapy, and 17 were excluded because they had ESRD and were on a regular dialysis program. The main clinical and demographic features are presented in Table 1. Of the 183 patients who constituted our sample, 115 (62.8%) were male and 162 (88.5%) were white. The median age was 65 (55–76) years. The most common reason for ICU admission was infection, accounting for 101 (57.0%) patients, and 132 (72.1%) were medical patients. The most common comorbidity was cardiovascular disease (n = 72; 39.3%), followed by hypertension (n = 60; 32.8%) and diabetes mellitus (n = 44; 24%). Sepsis was the most common cause of AKI (52.5%).

**Table 1:** Description of the main clinical and demographic data.

Variables	Number of cases (n = 183)
Gender	
Male	115 (62.8%)
Race	
Caucasian	162 (88.5%)
Age (years)	65 (55–76)
Patient classification	
Medical	132 (72.1%)
Surgical	51 (27.9%)
Reason for ICU admission	
Infection	101 (57.0%)
Cardiac failure	12 (6.5%)
Liver failure	12 (6.5%)
Hypovolemic/bleeding shock	2 (1.1%)
Other medical causes	5 (2.7%)
Solid organ transplantation	20 (10.9%)
(Liver transplantation)	15 (8.2%)
Elective or emergency surgery	31 (16.0%)
Comorbidities	
Diabetes mellitus	44 (24.0%)
Hypertension	60 (32.8%)
Cardiovascular disease	72 (39.3%)
Chronic obstructive pulmonary disease and other pulmonary diseases	23 (12.6%)
Solid organ neoplasia	28 (15.3%)
Hematologic malignancy or BMT	36 (19.6%)
Cirrhosis	38 (20.7%)
Liver transplantation	33 (18.0%)
Causes of AKI	
Sepsis	96 (52.5%)
Cardio renal syndrome	15 (8.2%)
Hepatorenal syndrome	16 (8.7%)
Solid organ transplantation	20 (10.9%)
Trauma and surgery	21 (11.5%)
Other causes	15 (8.2%)
Classification of AKI at beginning of CVVHDF	
KDIGO 3	120 (65.6%)
KDIGO 2	54 (29.5%)
KDIGO 1	9 (4.9%)
Baseline creatinine in mg/dL	1.00 (0.70–1.51)
Baseline eGFR in mL/min/1.73 m^2^	72 (40–102)
Vasopressors at beginning of CVVHDF	152 (83.0%)
Mechanical ventilation at beginning of CVVHDF	145 (79.2%)
Sedation at beginning of CVVHDF	136 (74.3%)
Accompanying oliguria at beginning of CVVHDF	114 (62.3%)
SAPS 3 score at ICU admission	61 (50–74)
Predicted mortality by SAPS 3 (%)	37.7 (20.1–64.0)
SOFA score at ICU admission	10 (7–12)
Total SOFA score on the first day of CVVHDF (n = 183)	13 (11–16)
Total SOFA score on the third day of CVVHDF (n = 173)	13 (11–15)
Total SOFA score on the seventh day of CVVHDF (n = 98)	14 (11–16)
Length of stay in hospital (days)	35 (21–67)
Length of stay in ICU (days)	14 (7–22)
Mortality up to 90 days	105 (57.4%)

*ICU*, intensive care unit; *BMT*, bone marrow transplantation; *AKI*, acute kidney injury; *CVVHDF*, continuous venovenous hemodiafiltration; e*GFR*, estimated glomerular filtration rate; *KDIGO*, Kidney Disease Improving Global Outcomes classification of acute kidney injury; *SAPS 3*, Simplified Acute Physiology Score 3; *SOFA*, Sequential Organ Failure Assessment score. Categorical variables described by absolute value (percentage) and numeric variables described by median (IQR).

On CVVHDF initiation, 152 patients (83%) were using vasoactive drugs, and 145 patients (79.2%) were on mechanical ventilation. The median SAPS 3 score at ICU admission was 61 (50–74), with a predicted mortality of 37.7% (20,1–64%). The SOFA score at ICU admission was 10 (7–12). The periods spent in hospital and in the ICU were 35 (21–67) and 14 (7–22), respectively.

The main data related to continuous dialysis, comparing survivors and non-survivors are presented in Table 2. The delivered dialysis dose was 33.2 ml/kg/h. The median time of CVVHDF was 6 days (3–10). The median time between hospital admission and CVVHDF initiation was 2 days (1–4), with no statistically significant difference between groups. The median time between ICU admission and CVVHDF initiation was 2 days (1–4). The median cumulative fluid balance during the CVVHDF period was -1838 ml (-5735 +2993). There median of interruption time during CVVHDF was 2 (0–10) hours. The number of patients who shifted from CVVHDF to intermittent dialysis was 68 (87,2%) in survivors group and 31 (29,5%) in non-survivors.

**Table 2:** Comparison of the aspects related to continuous dialysis between survivors and non-survivors.

Variables	Survivors (78)	Non-survivors (105)	Total (183)	P value
Dialysis prescribed dose (mL/kg/h)	37.6 (33–45.1)	38 (33.7–43.2)	37.9 (33.4–43.9)	0.86
Dialysis delivered dose (mL/kg/h)	32.8 (28.8–40)	33.6 (29.3–38.5)	33.2 (28.9–38.7)	0.88
Interruption time during CVVHDF (hours)	1 (0–8.6)	2 (0–15.8)	2 (0–10)	0,21
Number of days on CVVHDF	5 (3–8)	7 (3–12)	6 (3–10)	0.45
Time from ICU admission to starting time of CVVHDF (days)	1 (1–3)	2 (1–4)	2 (1–4)	0,01
Percentage change of weight from hospital admission to beginning of CVVHDF (%)	5.62 (0.82–12.13)	8.11 (0.95–17.97)	7.1 (1.0–15.4)	0.15
Fluid balance (cumulative) during dialysis (L)	-4.13 (-7.78–-0.77)	+0.65 (-3.68–+5.23)	-1.84 (-5.74–+ 2.99)	<0,001
Daily fluid balance during dialysis (L)	-0.74 (-1.28–-0.17)	-0.08 (-0.7–+1.0)	-0.35 (-1.08–+0.43)	<0,001

*CVVHDF*, continuous venovenous hemodiafiltration; *ICU*, intensive care unit. Categorical variables described by absolute value (percentage) and numeric variables described by median (IQR).

### Survival up to 90 days and factors related to death

The mortality rate observed during a 90-day period was 57.4% (n = 105).

Table 3 demonstrates the significant variables in the simple and multiple logistic regression analyses, using propensity score matching for positive daily fluid balance (yes or no). Kaplan Meier curve survival is described in S1 Fig. Cox regression analysis was also performed and it is described in S1 Table.

**Table 3:** Multivariate logistic regression with death at 90 days.

	Univariate analysis		Multivariate analysis	
	Odds ratio (CI 95%)	p-value	Odds ratio (CI 95%)	p-value
Positive daily fluid balance^a^ (yes vs no)	4.37 (2.15–8.88)	**<0.001**	4.55 (2.75–13.1)	**<0.001**
Infection	1.97 (1.09–3.60)	**0.025**		
COPD	2.32 (0.91–6.70)	**0.093**	3.44 (1.14–10.4)	**0.028**
Hematologic malignancy	2.82 (1.13–8.06)	**0.035**	5.14 (1.66–15.95)	**0.005**
Hepatic cirrhosis	2.47 (1.15–5.69)	**0.025**	2.39 (0.99–5.78)	**0.052**
Liver transplantation	0.41 (0.19–0.88)	**0.023**		
Vasopressors	2.12 (0.98–4.73)	**0.060**		
Mechanical ventilation	2.89 (1.39–6.18)	**0.005**		
Sedation	2.25 (1.15–4.46)	**0.018**		
Oliguria	1.85 (1.26–2.71)	**0.002**	2.36 (1.15–4.90)	**0.020**
SAPS 3 score	1.03 (1.01–1.05)	**0.002**		
Time from ICU admission to CVVHDF initiation (d)	1.07 (0.99–1.18)	**0.093**	1.10 (0.99–1.23)	**0.063**
SOFA	1.30 (1.15–1.46)	**<0.001**	1.27 (1.12–1.45)	**<0.001**

^a^ Treatment-effects estimation by propensity-score matching

*COPD*, chronic obstructive pulmonary disease; *CVVHDF*, continuous venovenoushemodiafiltration; *SAPS 3*, Simplified Acute Physiology Score 3

### Renal function at hospital discharge

Among the patients who survived (78) and those who recovered renal function (55 patients, 70.5% of survivors), the median baseline eGFR was 81 mL/min. (IQR: 47–115), higher than that observed in the group of dialysis-dependent patients at discharge (23 patients, 29.5% of survivors), in which the median was 38 mL/min (18 to 70), p = 0.002.

In those who recovered renal function, there was a reduction in eGFR at hospital discharge, to 50 ml/min (30–66) (p <0.001), and the median creatinine level was 1.4 mg/dl (1.0 to 1.9), when compared to the baseline.

### SOFA score

Regarding the SOFA scores, there were increased admission levels for day 1, and these remained higher than those on days 3 and 7, both for patients who died and for survivors (Table 4). However, when comparing the cardiovascular and respiratory SOFA components with fatal outcome, a statistically significant relationship was encountered, i.e., those who had died presented higher levels than the survivors on the first, third, and seventh days (Table 5).

**Table 4:** Comparisons of SOFA values in relation to admission.

SOFA score	Death	
	No	Yes
Admission in ICU	10 (7–12)	10 (7–12)
Day 1	12 (10–14)	14 (12–16)
Day 3	11 (9–14)	15 (13–17)
Day 7	10 (8–14)	15 (12–17)
p (Admission x Day 1)*	<0.001	<0.001
p (Admission x Day 3)*	<0.001	<0.001
p (Admission x Day 7)*	0.039	<0.001

*SOFA*, Sequential Organ Failure Assessment score; *ICU*, intensive care unit.

*Mann-Whitney´s test for non-parametric variable

**Table 5:** Comparison between cardiovascular and respiratory SOFA components on the first, third, and seventh days of dialysis and mortality.

Variables	Death		p-value*
	No	Yes	
Cardiovascular SOFA (mean ± SD)			
First day	2.8 ± 1.5	3.4 ± 1.1	0.005
Third day	2.0 ± 1.5	3.2 ± 1.2	< 0.001
Seventh day	1.7 ± 1.5	3.1 ± 1.4	< 0.001
Respiratory SOFA (mean ± SD)			
First day	1.4 ± 1.0	1.9 ± 1.1	0.004
Third day	1.2 ± 0.9	1.6 ± 1.1	0.009
Seventh day	1.0 ± 0.9	1.4 ± 1.1	0.069

*SOFA*, Sequential Organ Failure Assessment score

* Student’s t-test for the comparison of means

## Discussion

In this prospective observational study, critically ill patients with AKI, treated with CVVHDF, a positive fluid balance during dialysis was associated with shorter survival during the first 90 days.

Fluid overload causes stress to the cardiovascular system, edema in many tissues, and is associated with organ dysfunction and increased mortality rates [20–23]. Data suggest that the accumulation of fluid contributes to onset of AKI and seems to delay recovery time [24–26]. Venous congestion, increased intra-abdominal pressure, and increased renal interstitial pressure are possible pathophysiological mechanisms involved in the genesis and perpetuation of kidney damage [27,28].

Weight gain was observed in patients between hospital admission and the initiation of dialysis. Although some studies have showed that fluid overload, especially because volume resuscitation, is associated with higher mortality rates [29], the present data demonstrated that there was an increased risk of death in patients who were unable to achieve negative fluid balance during the period in which they were undergoing CRRT. According to our findings, in a cohort of 492 patients, Silversides et al. demonstrated that the most positive fluid balance after initiating RRT was associated with lower survival rates [11]. Analyzing data from the RENAL study with 1,453 patients, Bellomo et al. demonstrated that a negative fluid balance during RRT was associated with a lower risk of death as well as more free time for dialysis and shorter hospital and ICU stay [30].

It is a matter of debate whether the association between positive fluid balance and higher mortality rates is a result from a causal effect *per se* or only represents a disease severity marker [31]. By assessing the SOFA score, we observed that organ dysfunctions intensified during the period between ICU admission and the first week of dialysis. When specifically assessing the cardiovascular and respiratory SOFA components, it was observed that patients who died presented higher scores than those who survived during the first week of renal replacement therapy. Hypothetically, a more favorable hemodynamic profile could have provided greater volume withdrawal, thus decreasing pulmonary edema and improving the respiratory parameters of survivors. But, in our study the association remained after adjustment for confoudings, as SAPS3, SOFA score, and oliguria, suggesting that illness severity may not only account for our findings.

Another important aspect in AKI treatment is the time when dialysis is initiated. Theoretically, early initiation of dialysis should decrease the complications of fluid overload and the accumulation of uremic toxins. Moreover, it follows that patients who spontaneously recover their renal functions could have received dialysis unnecessarily [32–34]. In our cohort, the time between ICU admission and the onset of CVVHDF was not a risk factor for death.

Recently, two clinical trials addressed the important issue in the treatment of AKI. The first, a single-center study, demonstrated that the group of patients in which dialysis was initiated early (KDIGO stage 2) presented lower mortality rates after 90 days (39.3% vs. 54.7%) than the group who started renal replacement therapy later (KDIGO stage 3) [35]. The other, which was a multicenter study with a larger number of patients (n = 620), presented different results. When comparing early and late RRT (KDIGO 3, and at least one of the following criteria: hyperkalemia, metabolic acidosis, azotemia, pulmonary edema, or oliguria for more than 72 hours), there was no difference in mortality rates after 60 days (48.5% x 49.7%). Furthermore, in the group undergoing late renal replacement therapy, nearly 50% of patients recovered renal function and did not require dialysis [36]. The differences in the randomization criteria (stages of AKI) may explain these opposite results. In our sample, most of the patients were classified as KDIGO stage 3 and the AKI classification by this criterion was not associated with mortality. Two other large trials are ongoing, and the results will help to establish the best time to initiate renal replacement therapy in patients with AKI [37,38].

Until recently, it was believed that an intensive dose of dialysis affected the survival of patients with AKI in ICU. Ronco et al. reported that an effluent rate of 35–45 mL/kg/h increased the survival of patients with AKI undergoing continuous venovenous hemofiltration (CVVH) compared to a rate of 20 ml/kg/h [39]. Subsequently, two major studies, ATN and RENAL [40,41], determined that an intensive dose of dialysis (35–40 ml/kg/h) did not reduce the mortality of patients with AKI in the ICU and submitted to CVVHDF when compared to a dose of 20–25 ml/kg/h. In the present study, the effective effluent rate was around 30 ml/kg/h, which could be enough to counteract any interruptions in the procedures for filter change, tests, and surgical procedures.

Multivariate analysis revealed that oliguria was an independent risk factor for death. In more recent systems for defining and classifying AKI, the urine volume was included both as diagnostic criteria and for staging severity [42,43]. Some studies have also revealed that oliguria, regardless of creatinine levels, may be correlated with adverse outcomes, especially with the long-term need for dialysis and mortality [44].

Some comorbidities encountered in our patient population, specifically liver cirrhosis, hematologic malignancies, and COPD, were associated with a higher risk of death. Patients with COPD are susceptible to AKI, especially during periods of pulmonary disease exacerbation, and this association increases mortality. AKI is also a frequent complication and affects up to 2/3 of patients with hematologic malignancies admitted to ICUs. The etiology of AKI is multifactorial and is also an independent risk factor for death within this scenario [45–47].

Our study has several limitations. As this is an observational, single-center study, the population is subject to selection bias. There was no specific protocol for the choice of dialysis modality or for initiating RRT. Such decisions were left to the nephrology team, which, however, remained constant throughout the study period. There was also no protocol to define the removal rate of fluids during dialysis, and the desired fluid balance was planned daily after discussion between nephrologists and the ICU team.

## Conclusions

Dialysis-related factors may influence the outcomes in AKI. In our cohort, positive daily fluid balance during the period in which the patients remained in CVVHDF was associated with lower survival. Multicenter and randomized studies are needed to assess fluid balance as a primary outcome to define the best strategy in this patient population.

## Supporting information

S1 Table Cox regression univariate and multivariate analysis for 90-day mortality. (DOCX)

S1 Fig Kaplan-Meier survival curve for a 90-day period from CVVHDF initiation. *CVVHDF*, continuous venovenous hemodiafiltration.
(TIF)
