# Supplementary material for: Outcomes from a cohort of patients with acute kidney injury subjected to continuous venovenous hemodiafiltration: The role of negative fluid balance
Source: PLoS One. 2017 Apr 20;12(4):e0175897. doi: 10.1371/journal.pone.0175897 (PMC5398654; doi:10.1371/journal.pone.0175897)
Supplement: S1 Table — (DOCX) [file pone.0175897.s001.docx]

S1 Table – Cox regression univariate and multivariate analysis for 90-day mortality

|  | Univariate analysis | | Multiple analysis | |
| --- | --- | --- | --- | --- |
|  | Hazard ratio  (CI 95%) | p-value | Hazard ratio  (CI 95%) | p-value |
| COPD | 1.829 (1.087-3.078) | **0.023** | 1.83 (1.06-3.17) | 0.041 |
| Hematologic malignancy | 1.631 (1.002 – 2.657) | **0.049** |  |  |
| Hepatic cirrhosis | 1.54 (0.998-2.376) | **0.049** |  |  |
| Liver transplantation | 0.52 (0.29 - 0.93) | **0.029** |  |  |
| Vasopressors | 1.878 (1.05 – 3.359) | **0.031** |  |  |
| Mechanical ventilation | 2.243 (1.277 – 3.94) | **0.005** |  |  |
| Sedation | 1.785 (1.096 – 2.906) | **0.02** |  |  |
| Oliguria | 1.777 (1.168 - 2.705) | **0.007** | 1.671 (1.08 -2.58) | 0.018 |
| Fluid balance during CVVHDF (L) | 1.079(1.052 - 1.107) | **<0.001** | 1.08 (1.05-1.11) | <0,001 |
| SAPS 3 score | 1.021 (1.01 - 1.032) | **<0,001** | 1.02 (1.01-1.03) | 0.004 |
| Time from ICU admission to CVVHDF initiation | 1.04 (0.99 – 1.08) | **0.061** | 1.06 (1.02-1.1) | 0.004 |
| Percentual weight variation during CVVHDF (%) | 1.024 (1.004 – 1.044) | **0,02** |  |  |
| Absolut weight variation during CVVHDF (kg) | 1.028 (1.001-1.055) | **0.04** |  |  |
| Total SOFA (first dialysis day) | 2.17 (1.42-3.32) | **<0.001** | 1.72 (1.1-2.71) | 0.015 |

^a^SOFA score > 12 vs ≤ 12

*COPD,* chronic obstructive pulmonary disease; *CVVHDF,* continuous veno-venous hemodiafiltration; *SAPS 3,* Simplified Acute Physiology Score 3; *SOFA,* Sequential Organ Failure Assessment score

S1 Fig. Kaplan-Meier survival curve for a 90-day period from CVVHDF initiation. *CVVHDF*, continuous venovenous hemodiafiltration
