# Supplementary figures and images for: Outcomes from a cohort of patients with acute kidney injury subjected to continuous venovenous hemodiafiltration: The role of negative fluid balance
Source: PLoS One. 2017 Apr 20;12(4):e0175897. doi: 10.1371/journal.pone.0175897 (PMC5398654; doi:10.1371/journal.pone.0175897)

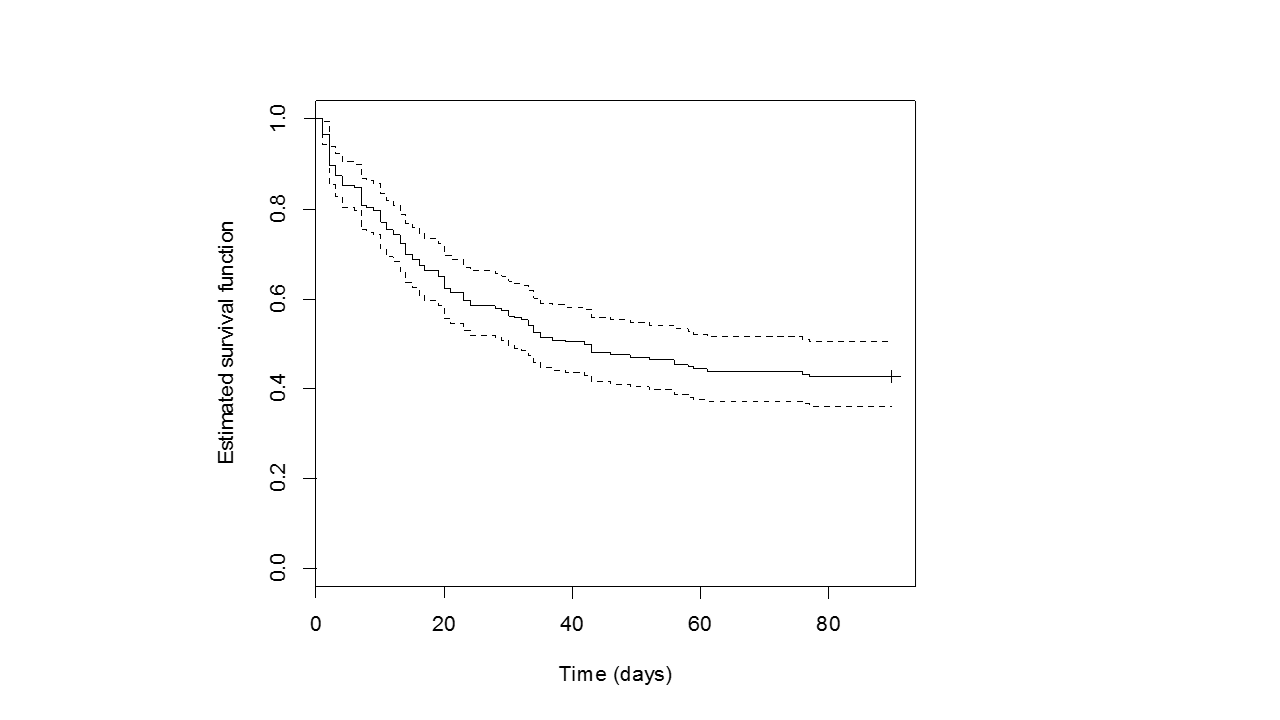

Supplement: S1 Fig — CVVHDF, continuous venovenous hemodiafiltration. (TIF) [file pone.0175897.s002.tif]
